# 4-Chloro-1-methyl­indoline-2,3-dione

**DOI:** 10.1107/S1600536811054171

**Published:** 2011-12-23

**Authors:** Jian Guang Yu, Wei Tang, De Cai Wang, Hong Xu

**Affiliations:** a State Key Laboratory of Materials-Oriented Chemical Engineering, College of Life Science and Pharmaceutical Engineering, Nanjing University of Technology, No. 5 Xinmofan Road, Nanjing 210009, People’s Republic of China; b State Key Laboratory of Materials-Oriented Chemical Engineering, College of Food Science and Light Industry, Nanjing University of Technology, No. 5 Xinmofan Road, Nanjing 210009, People’s Republic of China

## Abstract

The title mol­ecule, C_9_H_6_ClNO_2_, is essentially planar; the maximum deviation of the indoline ring system is 0.027 (3) Å and the substituents do not deviate by more than 0.075 (2) Å from this plane. Inter­molecular C—H⋯O hydrogen bonds consolidate the crystal structure.

## Related literature

For the preparation of the title compound, see: Bouhfid *et al.* (2005 ▶). For a related crystal structure and background to isatin derivatives, see: Liu *et al.* (2011 ▶).
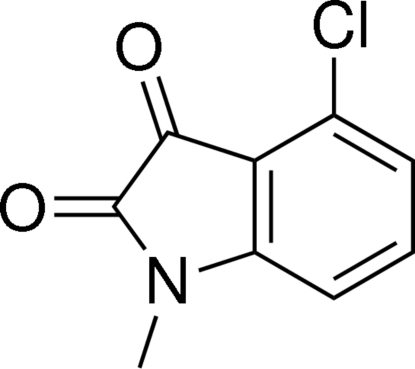


## Experimental

### 

#### Crystal data

C_9_H_6_ClNO_2_
*M* *_r_* = 195.60
Monoclinic, 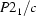

*a* = 7.4890 (15) Å
*b* = 14.825 (3) Å
*c* = 7.3140 (15) Å
β = 90.27 (3)°
*V* = 812.0 (3) Å^3^
*Z* = 4
Mo *K*α radiation
μ = 0.43 mm^−1^
*T* = 293 K
0.20 × 0.10 × 0.10 mm

#### Data collection

Enraf–Nonius CAD-4 diffractometer
Absorption correction: ψ scan (North *et al.*, 1968 ▶) *T*_min_ = 0.919, *T*_max_ = 0.958
1607 measured reflections
1485 independent reflections
965 reflections with *I* > 2σ(*I*)
*R*_int_ = 0.042
3 standard reflections every 200 reflections  intensity decay: 1%

#### Refinement

*R*[*F* ^2^ > 2σ(*F* ^2^)] = 0.060
*wR*(*F* ^2^) = 0.155
*S* = 1.00
1485 reflections
119 parameters
H-atom parameters constrained
Δρ_max_ = 0.28 e Å^−3^
Δρ_min_ = −0.28 e Å^−3^

### 

Data collection: *CAD-4 EXPRESS* (Enraf–Nonius, 1994 ▶); cell refinement: *CAD-4 EXPRESS*; data reduction: *XCAD4* (Harms & Wocadlo, 1995 ▶); program(s) used to solve structure: *SHELXS97* (Sheldrick, 2008 ▶); program(s) used to refine structure: *SHELXL97* (Sheldrick, 2008 ▶); molecular graphics: *SHELXTL* (Sheldrick, 2008 ▶); software used to prepare material for publication: *PLATON* (Spek, 2009 ▶).

## Supplementary Material

Crystal structure: contains datablock(s) global, I. DOI: 10.1107/S1600536811054171/pv2493sup1.cif

Structure factors: contains datablock(s) I. DOI: 10.1107/S1600536811054171/pv2493Isup2.hkl

Supplementary material file. DOI: 10.1107/S1600536811054171/pv2493Isup3.cml

Additional supplementary materials:  crystallographic information; 3D view; checkCIF report

## Figures and Tables

**Table 1:** Hydrogen-bond geometry (Å, °)

*D*—H⋯*A*	*D*—H	H⋯*A*	*D*⋯*A*	*D*—H⋯*A*
C2—H2*A*⋯O1^i^	0.93	2.58	3.323 (5)	137
C3—H3*A*⋯O2^i^	0.93	2.50	3.424 (5)	170
C9—H9*A*⋯O2^ii^	0.96	2.60	3.198 (5)	121

Symmetry codes: (i) 
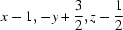
; (ii) 
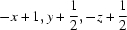
.

**Fig. 1.**
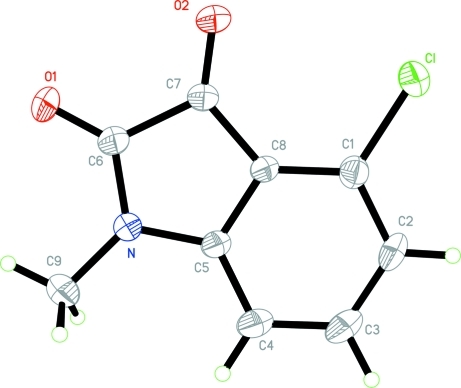
The molecular structure of the title molecule, showing the atom-numbering scheme and displacement ellipsoids at the 30% probability level.

**Fig. 2.**
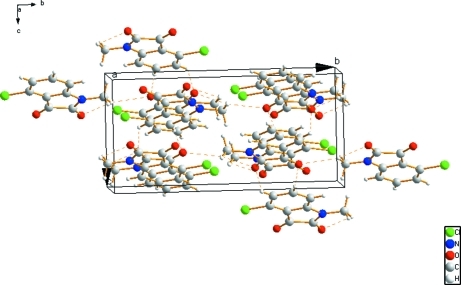
A packing diagram of the title compound. Intermolecular hydrogen bonds are shown as dashed lines.

## supplementary crystallographic information

### Comment

As a part of our studies on the synthesis and structures of isatin derivatives (Liu *et al.*, 2011), the title compound was synthesized and its structure is now reported in this article

The title molecule (Fig. 1) is essentially planar with the maximum deviation of C7 atom from the mean plane of the indoline ring (C1—C5/N/C6—C8) is 0.027 (3) A°. The substituents do not deviate more than 0.075 (2) Å from this plane. In the crystal structure, intermolecular and intramolecular C—H···O hydrogen bonds consolidate the crystal packing (Fig. 2 & Tab. 1).

### Experimental

The title compound was synthesized according to a reported procedure (Bouhfid *et al.*2005). 4-Chloroisatin (1.81 g, 0.01 mol) was reacted with iodomethane (2.84 g, 0.02 mol) in the presence of K_2_CO_3_ (2.76 g, 0.02 mol) and tetrabutylammonium bromide (0.32 g, 0.001 mol) in DMF (60 ml). After 12 h stirring at room temperature, the precipitate was removed by filtration and purified by recrystallization from ethanol (m.p. 464–467 K; yield 73%). Yellow crystals of the title compound were obtained by slow evaporation from ethanol at room temperature.

### Refinement

All H atoms were placed geometrically (C—H = 0.93–0.96 Å) and refined as riding with *U*_iso_(H) = 1.2*U*_eq_(aryl C) or 1.5*U*_eq_(methyl C).

### Crystal data

**Table:**

C_9_H_6_ClNO_2_	*F*(000) = 400
*M**_r_* = 195.60	*D*_x_ = 1.600 Mg m^−^^3^
Monoclinic, *P*2_1_/*c*	Mo *K*α radiation, λ = 0.71073 Å
Hall symbol: -P 2ybc	Cell parameters from 25 reflections
*a* = 7.4890 (15) Å	θ = 9–12°
*b* = 14.825 (3) Å	µ = 0.43 mm^−^^1^
*c* = 7.3140 (15) Å	*T* = 293 K
β = 90.27 (3)°	Block, yellow
*V* = 812.0 (3) Å^3^	0.20 × 0.10 × 0.10 mm
*Z* = 4	

### Data collection

**Table:**

Enraf–Nonius CAD-4 diffractometer	965 reflections with *I* > 2σ(*I*)
Radiation source: fine-focus sealed tube	*R*_int_ = 0.042
graphite	θ_max_ = 25.5°, θ_min_ = 2.7°
ω/2θ scans	*h* = −9→9
Absorption correction: ψ scan (North *et al.*, 1968)	*k* = 0→17
*T*_min_ = 0.919, *T*_max_ = 0.958	*l* = 0→8
1607 measured reflections	3 standard reflections every 200 reflections
1485 independent reflections	intensity decay: 1%

### Refinement

**Table:**

Refinement on *F*^2^	Secondary atom site location: difference Fourier map
Least-squares matrix: full	Hydrogen site location: inferred from neighbouring sites
*R*[*F*^2^ > 2σ(*F*^2^)] = 0.060	H-atom parameters constrained
*wR*(*F*^2^) = 0.155	*w* = 1/[σ^2^(*F*_o_^2^) + (0.075*P*)^2^] where *P* = (*F*_o_^2^ + 2*F*_c_^2^)/3
*S* = 1.00	(Δ/σ)_max_ < 0.001
1485 reflections	Δρ_max_ = 0.28 e Å^−^^3^
119 parameters	Δρ_min_ = −0.28 e Å^−^^3^
0 restraints	Extinction correction: *SHELXL97* (Sheldrick, 2008), Fc^*^=kFc[1+0.001xFc^2^λ^3^/sin(2θ)]^-1/4^
Primary atom site location: structure-invariant direct methods	Extinction coefficient: 0.024 (4)

### Special details

**Table:**

Geometry. All e.s.d.'s (except the e.s.d. in the dihedral angle between two l.s. planes) are estimated using the full covariance matrix. The cell e.s.d.'s are taken into account individually in the estimation of e.s.d.'s in distances, angles and torsion angles; correlations between e.s.d.'s in cell parameters are only used when they are defined by crystal symmetry. An approximate (isotropic) treatment of cell e.s.d.'s is used for estimating e.s.d.'s involving l.s. planes.
Refinement. Refinement of *F*^2^ against ALL reflections. The weighted *R*-factor *wR* and goodness of fit *S* are based on *F*^2^, conventional *R*-factors *R* are based on *F*, with *F* set to zero for negative *F*^2^. The threshold expression of *F*^2^ > σ(*F*^2^) is used only for calculating *R*-factors(gt) *etc*. and is not relevant to the choice of reflections for refinement. *R*-factors based on *F*^2^ are statistically about twice as large as those based on *F*, and *R*- factors based on ALL data will be even larger.

### Fractional atomic coordinates and isotropic or equivalent isotropic displacement parameters (Å^2^)

**Table:**

	*x*	*y*	*z*	*U*_iso_*/*U*_eq_	
Cl	0.19910 (14)	0.55890 (7)	0.16100 (16)	0.0561 (4)	
N	0.3254 (4)	0.88873 (19)	0.2061 (4)	0.0430 (8)	
O1	0.6058 (4)	0.87503 (19)	0.3282 (5)	0.0676 (10)	
C1	0.1324 (5)	0.6685 (2)	0.1324 (5)	0.0425 (9)	
O2	0.5204 (4)	0.68317 (18)	0.3142 (4)	0.0606 (9)	
C2	−0.0323 (5)	0.6886 (3)	0.0589 (5)	0.0490 (11)	
H2A	−0.1085	0.6423	0.0230	0.059*	
C3	−0.0848 (5)	0.7773 (3)	0.0381 (6)	0.0540 (12)	
H3A	−0.1982	0.7897	−0.0078	0.065*	
C4	0.0265 (5)	0.8479 (3)	0.0835 (6)	0.0484 (10)	
H4A	−0.0097	0.9074	0.0677	0.058*	
C5	0.1921 (5)	0.8279 (2)	0.1525 (5)	0.0375 (9)	
C6	0.4690 (5)	0.8435 (3)	0.2721 (6)	0.0470 (10)	
C7	0.4229 (5)	0.7422 (2)	0.2603 (5)	0.0412 (9)	
C8	0.2447 (4)	0.7390 (2)	0.1784 (5)	0.0370 (9)	
C9	0.3123 (5)	0.9847 (3)	0.1887 (7)	0.0587 (12)	
H9A	0.4200	1.0121	0.2338	0.088*	
H9B	0.2126	1.0060	0.2584	0.088*	
H9C	0.2957	1.0003	0.0624	0.088*	

### Atomic displacement parameters (Å^2^)

**Table:**

	*U*^11^	*U*^22^	*U*^33^	*U*^12^	*U*^13^	*U*^23^
Cl	0.0597 (7)	0.0389 (6)	0.0696 (8)	−0.0050 (5)	−0.0120 (5)	−0.0014 (5)
N	0.0351 (18)	0.0338 (17)	0.060 (2)	0.0011 (14)	−0.0083 (15)	−0.0031 (16)
O1	0.0420 (17)	0.0512 (18)	0.109 (3)	−0.0072 (14)	−0.0290 (17)	−0.0120 (18)
C1	0.043 (2)	0.042 (2)	0.043 (2)	−0.0048 (17)	−0.0040 (18)	−0.0005 (18)
O2	0.0446 (17)	0.0455 (17)	0.092 (2)	0.0107 (13)	−0.0241 (16)	−0.0012 (16)
C2	0.036 (2)	0.063 (3)	0.048 (2)	−0.016 (2)	−0.0084 (19)	−0.003 (2)
C3	0.034 (2)	0.077 (3)	0.051 (2)	0.000 (2)	−0.0105 (19)	0.006 (2)
C4	0.036 (2)	0.056 (2)	0.052 (2)	0.0071 (19)	−0.0058 (18)	0.006 (2)
C5	0.033 (2)	0.039 (2)	0.041 (2)	0.0036 (16)	−0.0057 (16)	−0.0009 (17)
C6	0.033 (2)	0.043 (2)	0.065 (3)	0.0051 (18)	−0.0069 (19)	−0.005 (2)
C7	0.034 (2)	0.037 (2)	0.052 (2)	0.0065 (17)	−0.0039 (18)	−0.0021 (19)
C8	0.0305 (19)	0.035 (2)	0.045 (2)	0.0036 (15)	−0.0068 (17)	−0.0031 (17)
C9	0.057 (3)	0.035 (2)	0.083 (3)	0.002 (2)	−0.006 (2)	0.001 (2)

### Geometric parameters (Å, °)

**Table:**

Cl—C1	1.712 (4)	C3—C4	1.378 (6)
N—C6	1.354 (5)	C3—H3A	0.9300
N—C5	1.400 (4)	C4—C5	1.369 (5)
N—C9	1.431 (5)	C4—H4A	0.9300
O1—C6	1.197 (4)	C5—C8	1.389 (5)
C1—C2	1.376 (5)	C6—C7	1.544 (5)
C1—C8	1.382 (5)	C7—C8	1.461 (5)
O2—C7	1.204 (4)	C9—H9A	0.9600
C2—C3	1.381 (6)	C9—H9B	0.9600
C2—H2A	0.9300	C9—H9C	0.9600
			
C6—N—C5	110.2 (3)	C8—C5—N	111.8 (3)
C6—N—C9	125.2 (3)	O1—C6—N	127.3 (4)
C5—N—C9	124.6 (3)	O1—C6—C7	126.1 (4)
C2—C1—C8	118.3 (4)	N—C6—C7	106.6 (3)
C2—C1—Cl	120.9 (3)	O2—C7—C8	131.4 (4)
C8—C1—Cl	120.7 (3)	O2—C7—C6	123.6 (3)
C1—C2—C3	120.2 (4)	C8—C7—C6	104.9 (3)
C1—C2—H2A	119.9	C1—C8—C5	120.8 (3)
C3—C2—H2A	119.9	C1—C8—C7	132.7 (3)
C4—C3—C2	121.7 (4)	C5—C8—C7	106.5 (3)
C4—C3—H3A	119.2	N—C9—H9A	109.5
C2—C3—H3A	119.2	N—C9—H9B	109.5
C5—C4—C3	118.0 (4)	H9A—C9—H9B	109.5
C5—C4—H4A	121.0	N—C9—H9C	109.5
C3—C4—H4A	121.0	H9A—C9—H9C	109.5
C4—C5—C8	120.8 (4)	H9B—C9—H9C	109.5
C4—C5—N	127.4 (4)		
			
C8—C1—C2—C3	−2.2 (6)	N—C6—C7—O2	−177.4 (4)
Cl—C1—C2—C3	179.5 (3)	O1—C6—C7—C8	−178.4 (4)
C1—C2—C3—C4	2.3 (6)	N—C6—C7—C8	1.3 (4)
C2—C3—C4—C5	−0.7 (6)	C2—C1—C8—C5	0.6 (6)
C3—C4—C5—C8	−1.0 (6)	Cl—C1—C8—C5	178.9 (3)
C3—C4—C5—N	179.6 (4)	C2—C1—C8—C7	178.7 (4)
C6—N—C5—C4	178.3 (4)	Cl—C1—C8—C7	−3.0 (6)
C9—N—C5—C4	−3.1 (6)	C4—C5—C8—C1	1.0 (6)
C6—N—C5—C8	−1.1 (5)	N—C5—C8—C1	−179.5 (3)
C9—N—C5—C8	177.4 (4)	C4—C5—C8—C7	−177.5 (4)
C5—N—C6—O1	179.5 (4)	N—C5—C8—C7	1.9 (4)
C9—N—C6—O1	0.9 (7)	O2—C7—C8—C1	−1.7 (8)
C5—N—C6—C7	−0.2 (4)	C6—C7—C8—C1	179.8 (4)
C9—N—C6—C7	−178.7 (4)	O2—C7—C8—C5	176.6 (4)
O1—C6—C7—O2	3.0 (7)	C6—C7—C8—C5	−1.9 (4)

### Hydrogen-bond geometry (Å, °)

**Table:**

*D*—H···*A*	*D*—H	H···*A*	*D*···*A*	*D*—H···*A*
C2—H2A···O1^i^	0.93	2.58	3.323 (5)	137
C3—H3A···O2^i^	0.93	2.50	3.424 (5)	170
C9—H9A···O1	0.96	2.56	2.915 (5)	102
C9—H9A···O2^ii^	0.96	2.60	3.198 (5)	121

Symmetry codes: (i) *x*−1, −*y*+3/2, *z*−1/2;  (ii) −*x*+1, *y*+1/2, −*z*+1/2.
